# PELI3 mediates pro-tumor actions of down-regulated miR-365a-5p in non-small cell lung cancer

**DOI:** 10.1186/s40659-019-0230-y

**Published:** 2019-04-17

**Authors:** Yuzheng He, Yantao Shi, Ruilin Liu, Zhichao Wang, Baohua Wang, Shujun Li, Helin Zhang

**Affiliations:** Department of Thoracic Surgery, The Second Hospital of Hebei Medical University, No. 215 Heping West Road, Shijiazhuang, 050000 Hebei China

**Keywords:** PELI3, miR-365a-5p, Non-small cell lung cancer, Cell viability

## Abstract

**Background:**

To analyze the relative expression of PELI3 and its mechanistic involvement in the non-small cell lung cancer (NSCLC).

**Methods:**

PELI3 expression in NSCLC tissue samples was determined by the immunohistochemistry. The transcripts abundance of PELI3 was measured with real-time PCR. The protein intensity was analyzed by western blot. The overall survival in respect to PELI3 or miR-365a-5p expression was plotted by the Kaplan–Meier’s analysis. Cell growth was determined by colony formation assay. Cell viability was measured by MTT assay. The migration and invasion were evaluated by wound healing and transwell assay respectively. The regulatory effect of miR-365a-5p on PELI3 was interrogated with luciferase reporter assay. The direct binding between miR-365a-5p and PELI3 was analyzed by pulldown assay.

**Results:**

PELI3 was aberrantly up-regulated in NSCLC both in vivo and in vitro. High level of PELI3 associated with poor prognosis. PELI3-deficiency significantly inhibited cell viability, colony formation, migration and invasion. We further identified that miR-365a-5p negatively regulated PELI3 in this disease. Ectopic expression of miR-365a-5p in both A549 and H1299 phenocopied PELI3-deficiency. Meanwhile, PELI3-silencing significantly abolished the pro-tumoral effect elicited by miR-365a-5p inhibition.

**Conclusion:**

Our results highlighted the importance of dysregulated miR-365a-5p-PELI3 signaling axis in NSCLC.

## Introduction

Lung cancer is the most common human malignancy and the first cause of cancer-related death in men and second after breast cancer in women [1]. In 2012, 1.82 million new cases were diagnosed worldwide and 1.56 million deaths were claimed which accounting for around 20% of all deaths from cancer [2]. Lung cancer are conventionally classified by the histological types and two broad classes are distinguished including non-small-cell lung carcinoma (NSCLC) and small-cell lung carcinoma [3]. The most frequent risk factor related to the vast majority of lung cancer is long-term tobacco smoking [4]. In the never smoker, the incidence of lung cancer is often due to the combinational influences of genetic abnormalities with occupational exposure to radon gas, asbestos, smoke and air pollution [5]. Clinical options and end-point outcomes for this disease heavily depend on the type of cancer, progression and overall health conditions, while most cases are not curable. The mainstay therapeutics includes surgery, chemotherapy, radiotherapy, and more recently, the targeted therapies [6]. However, the 5-year survival of NSCLC is relatively unfavorable and only 11.2% patients survive this disease. The clinical benefits of targeted therapies are greatly promising in expansion of the survival period, which are currently only applicable to those patients with specified genetic mutations in either epidermal growth factor receptor (EGFR) or receptor activator of nuclear factor kappa-B ligand (RANKL) [7]. Despite of the tremendous advance in understanding the etiology of NSCLC, the molecular mechanism underlying the incidence and metastasis of some cases NSCLS are still elusive and to be precisely defined, which definitely would offer the chance for early diagnostic and therapeutic purpose.

Pellino E3 ubiquitin protein ligase family member 3 (PELI3) was initially identified as as a scaffolding protein which promotes activation of c-Jun and Elk-1 [8] and an intermediate signaling protein in the innate immune response pathway. The subsequent investigations characterized the ligase activity and ubiquitin-protein transferase activity against IRAK1 [9]. The PELI3 protein has also been reported to facilitate the transmission of the immune response signaling from Toll-like receptors to IRAK/TRAF6 complex [10]. Autophagy-dependent PELI3 degradation inhibits proinflammatory IL1B expression [11]. Notably, the potential involvement of PELI3 has been barely investigated until now.

microRNAs are defined as small oligonucleotides with average length between 18 and 25 base pairs and highly evolutional conservation [12]. microRNAs exert their biological functions via recognition and binding to the 3′UTR region and post-transcriptionally modulated target gene expression. With advance in the assembling experimental evidences, microRNAs have been identified indispensable in multiple biological processing such as mRNA stability and translation. Dysregulation in micorRNAs therefore frequently links to variety of human disease including hereditary progressive hearing loss, skeletal and growth defects, cancer, kidney disease, alcoholism and obesity [13]. Multiple dimensional effects of microRNAs have been increasingly uncovered in human malignancies as well involving in cell proliferation, cell cycle, apoptosis, angiogenesis, cell migration and invasion [14].

Here we set out to characterize the expression pattern of PELI3 in NSCLC and elucidate its mechanistic involvement in the initiation and progression of this disease. Our study will offer better understanding into the relevance of aberrantly regulated PELI3 with NSCLC.

## Materials and methods

### Clinical patients

The non-small cell lung cancer patients were enrolled in the Second Hospital of Hebei Medical University in this study with written informed consents. This study was approved by the Ethics Committee of the Second Hospital of Hebei Medical University. All tumors were pathologically confirmed by at least three experienced experts.

### Cell culture

The human non-small cell lung cancer cell lines A549, SPCA1, H1299, H358, PC9 and immortalized human bronchial epithelial cell 16HBE were obtained from the American Type Culture Collection (ATCC, VA, USA). The cell identities and mycoplasma-free were authenticated by DNA profiling. All cancer cells were maintained in RPMI modified medium (Gibco, Grand Island, NY) supplemented with 10% fetal bovine serum and 1% penicillin/streptomycin. HBEC cell was kept in Airway Epithelial Cell Basal Medium supplemented with the Bronchial Epithelial Cell Growth Kit (ATCC, VA, USA). Cell culture was performed in the humidified CO_2_ (5%) incubator. Exponentially growing cells were used for the experimental analysis. Lipofectamine 2000 (Invitrogen, MA, USA) was used for cell transfection purpose. Transfection efficacy was evaluated with GFP. For transient knockdown, siRNAs were synthesized by the Ribobio (Guangzhou, China).

### Real-time PCR

Trizol reagent (Invitrogen, Waltham, MA USA) was employed to extract total RNA from lung cancer cells. The RNA samples were quality-checked by agarose electrophoresis and quantified with Nanodrop 2000 (Invitrogen, Waltham, MA USA). The cDNA was prepared from each 1 μg RNA using the First Strand cDNA Synthesis Kit (Sigma, St. Louis, MO) following the manufacturer’s guide. For miR quantitative purpose, reverse transcription was performed with miScript II RT Kit (Qiagen, Valencia, CA, USA). Real-time PCR was performed with the SYBR Green Real-Time PCR Master Mixes (ThermoFisher, Waltham, MA USA). The endogenous GAPDH was employed as internal control. For real-time PCR with miR, the miScript SYBR Green PCR Kit (Qiagen) was used. U6 was employed as internal control for miR quantitation. The primers were provided as below:

- PELI3 Forward primer: CTGGAAGGAAACCCTGAAGT
- PELI3 Reverse primer: AGCGGCGTGGAGATGTG,
- GAPDH Forward primer: ACAACTTTGGTATCGTGGAAGG;
- GAPDH Reverse primer: GCCATCACGCCACAGTTTC.
- miR-365a-5p reverse transcription primer: GTCGTATCCAGTGCAGGGTCCGAGGTATTCGCACTGGATACGACCACATC;
- miR-365a-5p Forward primer: CTGAGGGACTTTTGGGGGCAG,
- miR-365a-5p Reverse primer: GTGCAGGGTCCGAGGT;
- U6 reverse transcription primer: GTCGTATCCAGTGCAGGGTCCGAGGTATTCGCACTGGATACGACAAAATATGGAA.
- U6 Forward primer: TGCGGGTGCTCGCTTCGGCAGC,
- U6 Reverse primer: GTGCAGGGTCCGAGGT.

### 3-(4,5-Dimethylthiazol-2-yl)-2,5-diphenyltetrazolium bromide (MTT) assay

Cell viability was evaluated by the MTT method. The commercially available MTT Cell Proliferation Assay Kit (Cayman, Ann Arbor, MI, USA) was adopted for this assay. Briefly, transient PELI3-knockdown A549 and H1299 cells (1000 cells/well) were seeded into 96-well plate in triplicate and cultured for 24 h. 10 μL MTT solution was added into each well and incubated the plate in the CO_2_ chamber for 3 h to allow the formazan formation. Dissolution was conducted with 100 μL of dissolving solution and incubated for another 4 h. The absorption was measured at 570 nm with 490 nm as reference.

### Western blot

The indicated cells were lysed in ice-cold RIPA buffer (Beyotime, Nantong, China) for 30 min. The cell debris was discarded after refrigerated high-speed centrifugation (12,000 rpm by 15 min). The protein concentration was measured by the BCA method (Bicinchoninic Acid Kit, Sigma). The protein samples were first separated by the SDS-PAGE followed by the PVDF membrane transfer. The membranes were subjected to non-fat milk blocking, primary antibody incubation and secondary hybridization consecutively. The protein blots were visualized by the enhanced chemiluminescence kit (ECL Substrate Kit, Abcam, Cambridge, UK).

### Immunohistochemistry

The fresh or flash-frozen in liquid nitrogen tissue samples were dehydrated first and followed by the fixation and paraffin embedding. Fixed and embedded in paraffin. The tissue blocks were cut into 5 μΜ sections and followed by the deparaffinization and rehydration. Antigen retrieval was performed with sodium citrate (pH 6.0) and boiled in microwave for 20 min. The endogenous peroxidase was blocked with 5% normal goat serum. The primary antibody (1:100) was incubated at 4 °C overnight. After rinsing with tris-buffered saline and Tween 20 buffer and H_2_O_2_ incubation, horseradish peroxidase-conjugated secondary antibody was applied for 1 h at room temperature. The protein was visualized with DAB (Boster, Wuhan, China).

### Colony formation

Cell proliferation in response to PELI3 deficiency was determined by the colony formation assay. 1000 exponentially growing cells were seeded into 6-well plate in triplicate. Consecutive cell culture was performed in CO_2_ hood for 2 weeks. The formed colonies were fixed with methanol and stained with 0.25% crystal violet.

### Transwell chamber

Cell invasive capacity was determined with Basal Membrane Extract (BD BioSciences, CA, USA)-precoated Transwell chambers (Corning, NY, USA). The single-cell suspension (20,000 cells/100 μL) was prepared in serum-free medium and laid on the upper insert. The complete culture medium (650 μL) was filled into the bottom compartment. After 12 h, the carbonate filter was cautiously removed and subjected to fixation and staining with crystal violet.

### Wound healing

The migrative capacity of indicated cells was determined by the wound healing assay. The log phase single layer cells were cultured in 6-well plate overnight. The straight scratch of uniform width was created with sterile tips. The closure of scratch was continuously monitored under light microscope.

### Luciferase reporter assay

The PELI3 3′UTR region was sub-cloned into pGL4 dual luciferase reporter vectors with the following primers:

- WT-F: CTACTCGAGGCTCCCTGGGGCCCCC,
- WT-R: CTAGCGGCCGCTTCTGGAGAGTGCTCAATGGA;
- Mut-F: CTCCGGTCGCGCCACATGCCGGA,
- Mut-R: TCCGGCATGTGGCGCGACCGGAG.

Either wild-type or mutated luciferase reporter plasmids co-transfected into A549 and H1299 cells with miR-365a-5p. After 24 h, the relative luciferase activity was measured with Luciferase Assay System (Promega, WI, USA) in accordance with the manufacturer’s instruction.

### Pulldown assay

The direct binding between miR-365a-5p and PELI3 transcript was interrogated by the pulldown assay. Biotin-labelled miR-365a-5p was purchase from Ribobio (Guangzhou, China). The cell lysate was prepared from both A549 and H1299 cell lines, which was subsequently incubated with biotin-miR-365a-5p at 4 °C for 2 h. The mixture was then pulled down by the Streptavidin-couple Dynabeads (ThermoFisher). After elution, the enriched PELI3 transcripts were quantified by the real-time PCR.

### Xenograft tumor model

To establish xenograft tumor model, PELI3 was stably silenced in A549 by shRNAs, which was subjected to puromycin (2 μg/mL) selection and knockdown efficiency was confirmed by real-time PCR. BALB/c nude mice (4-week) were purchased from the Vital River (Beijing, China) and housed in the specific pathogen-free environment with free access to drinking water and food. 1 × 10^6^ cells resuspended in serum-free medium was mixed with equal volume of Matrigel (BD BioSciences) and subcutaneously inoculated into right flank of nude mice. The tumor growth was monitored regularly until the size approaching 1500 mm^3^ and subjected to sacrifice. The xenograft tumor was resected and representative macroscopic images were captured. For lung metastasis evaluation, 5 × 10^5^ cells were tail vein injected and lung nodules were determined by H&E staining.

### Statistical analysis

All data were acquired from at least three independent experiments. GraphPad PRISM 6.0 software was used for data analysis and processing. Statistical comparison was performed with ANOVA followed the Student *t*-test. The *p* value was calculated and *p* < 0.05 was considered as significantly different.

## Results

### The expression of PELI3 is up-regulated in NSCLC and increased PELI3 expression predicts poor prognosis in NSCLC patients

**Fig. 1:**
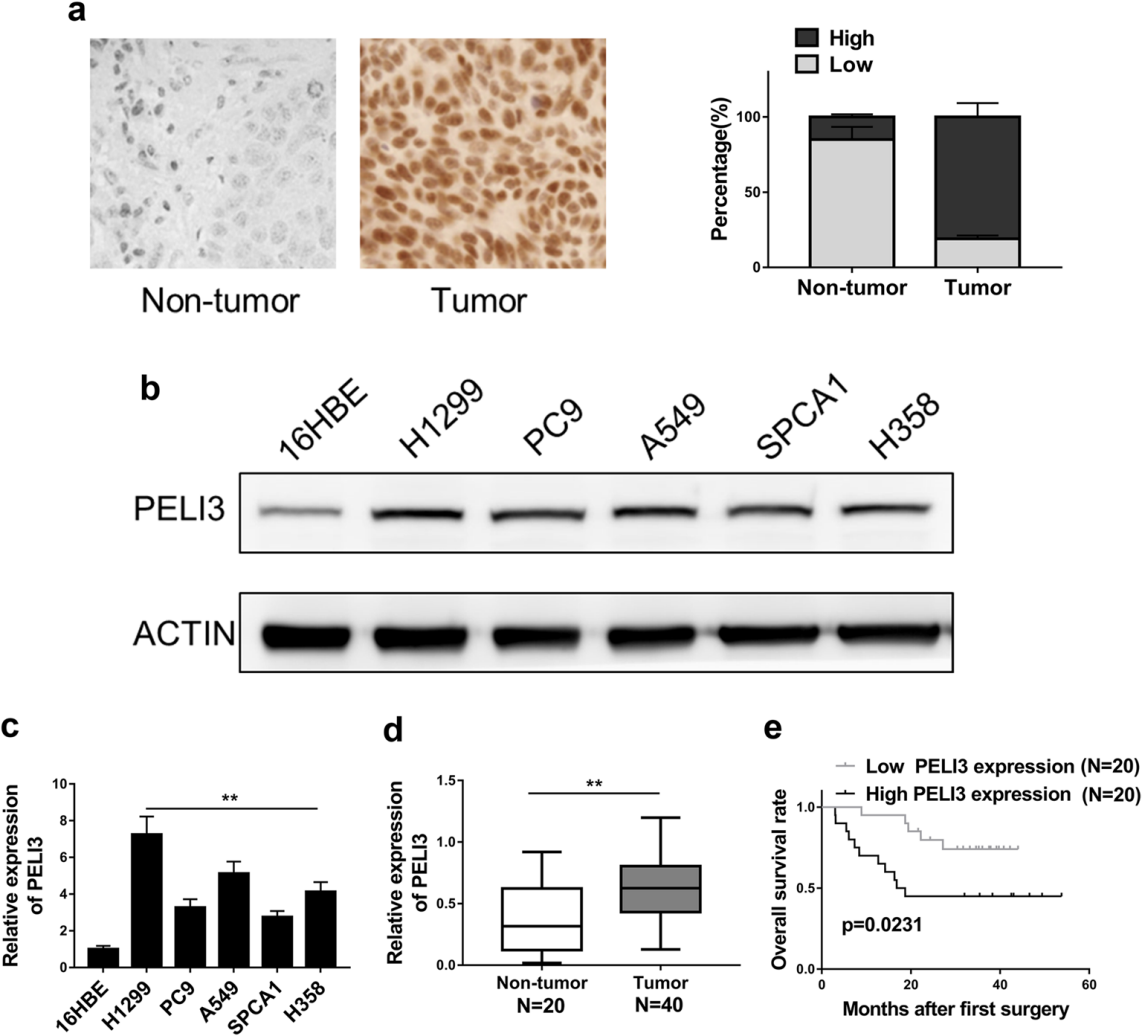
The expression of PELI3 is up-regulated in NSCLC and increased PELI3 expression predicts poor prognosis in NSCLC patients. **a** IHC analysis of PELI3 expression in NSCLC tissue samples and non-tumor tissue samples, 400X. **b**, **c** The expression levels of PELI3 in NSCLC cells (A549, SPCA1, H1299, H358, PC9) and human bronchial epithelial cells (16HBE) were detected by western blot and qRT-PCR. **d** The PELI3 expression levels in 40 NSCLC tissues and 20 non-tumor tissues were detected by qRT-PCR. **e** Kaplan–Meier’s analysis of the correlation between PELI3 expression and the overall survival rate of NSCLC patients. The data represent the mean ± SD from three independent experiments. *P < 0.05; **P < 0.01

We first determined the relative expression of PELI3 in NSCLC clinical tissue samples; the significantly intensive signal was detected in the tumor samples in comparison with the benign control (Fig. 1a). The aberrant up-regulation of PELI3 was further confirmed in the cell culture. The relative high expression of PELI3 protein was observed in all tested NSCLC cell lines including H1299, PC9, A549, SPCA1 and H358 compared to the immortalized human bronchial epithelial cell 16HBE (Fig. 1b). The most remarkable overexpression of PELI3 was detected in A549 and H1299 cells, which were then chosen for the following study unless specified. Likewise, the mRNA level of PELI3 was up-regulated as well experimentally validated in the real-time PCR (Fig. 1c). The in vivo transcripts of PELI3 in clinical samples were also shown much higher in tumor than in normal control (Fig. 1d). In support of the potential oncogenic role of PELI3 in NSCLC, our Kaplan–Meier’s analysis demonstrated the more favorable prognosis associated with low PELI3 level in the NSCLC patients (Fig. 1e). In our clinical sample pool, we observed 17.6% PELI3-positivity in early stage of this disease while 73.9% of all cases were PELI3-positive in stage III–IV NSCLC (p = 0.0011) (Table 1). Therefore, we provided evidences that PELI3 was over-expressed in NSCLC both in vivo and in vitro and indicated the potential pro-tumoral property of PELI3.

**Table 1:** Correlation of PELI3 expression with tumor stage in NCSLC

Expression of PELI3			
Parameter	Low (%)	High (%)	P value
Tumor stage			0.0011
I–II	14 (82.4%)	3 (17.6%)	
III–IV	6 (26.1%)	17 (73.9%)	

### Knockdown of PELI3 inhibits proliferation, migration and invasion of NSCLC cell lines

**Fig. 2:**
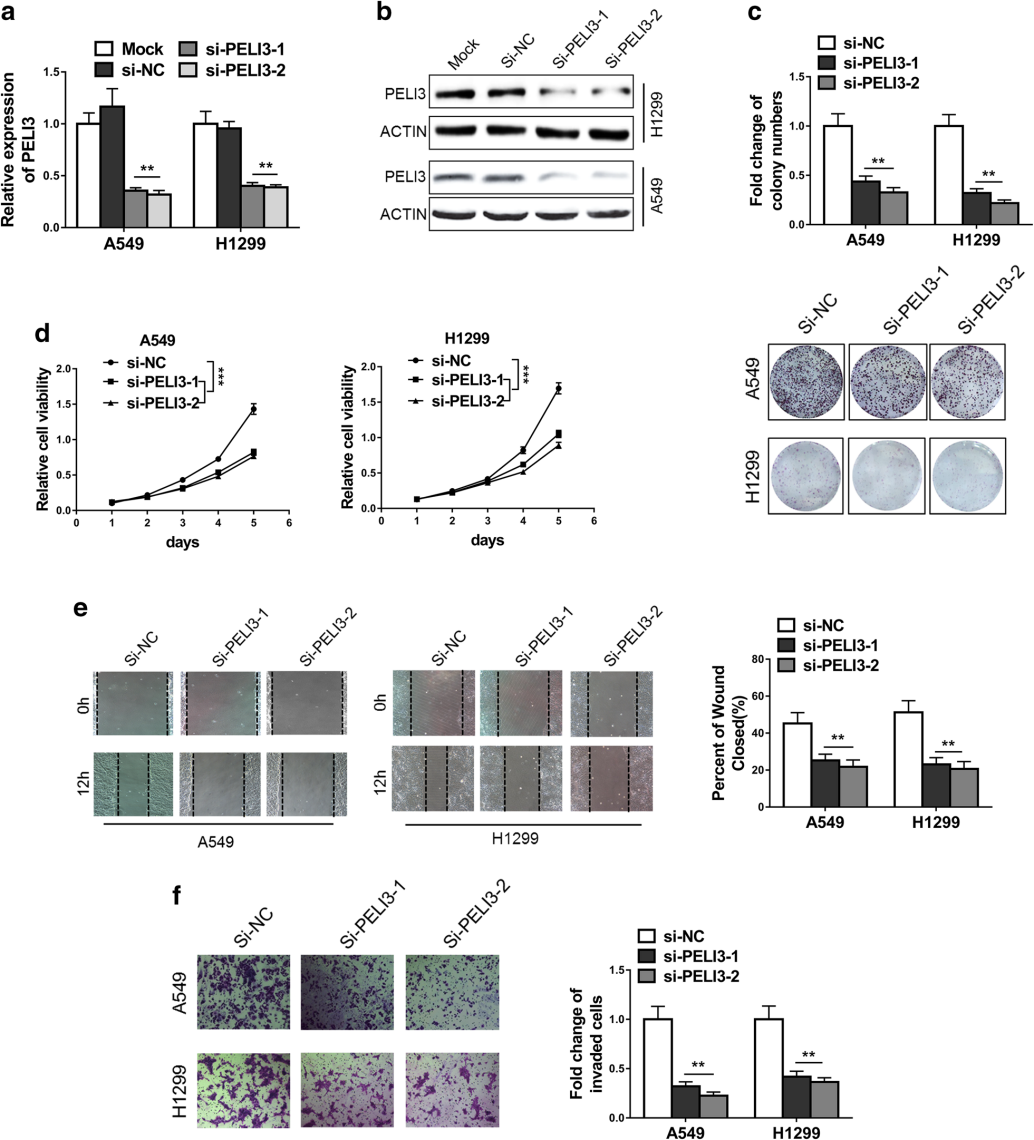
Knockdown of PELI3 inhibits proliferation, migration and invasion of NSCLC cell lines. **a**, **b** Knockdown efficiency of PELI3 in A549 and H1299 cells transfected with PELI3 siRNA (si-PELI3-1 and si-PELI3-2) or negative control siRNA (si-NC) was confirmed by qRT-PCR and western blot. **c**, **d** Colony formation and MTT assays showed knockdown of PELI3 significantly suppressed proliferation of A549 and H1299 cells. **e** Wound-healing assays showed the repression of cell migration of A549 and H1299 due to knockdown of PELI3. **f** Transwell assays showed the repression of cell invasion of A549 and H1299 due to knockdown of PELI3. The data represent the mean ± SD from three independent experiments. *P < 0.05; **P < 0.01; ***P < 0.001 (two-way ANOVA for d, student’s t-test for others)

To experimentally validate the oncogenic role of PELI3 in NSCLC, here we employed two independent siRNAs to transiently knockdown PELI3 in both A549 and H1299. The knockdown efficacies were confirmed at both transcript (Fig. 2a) and protein (Fig. 2b) levels in comparison with either mock or scramble controls. The potential impacts on the cell growth and malignant behaviors were determined in response to PELI3 silencing. As shown in Fig. 2c, the colony formation capacity was significantly compromised in the PELI3-deficient A549 and H1299 cells in comparison with the PELI3-proficient counterparts. The representative images were provided in the lower panel for reference. Similarly, the cell viability was remarkably inhibited upon PELI3 knockdown in both A549 and H1299 cells (Fig. 2d). In addition, the migrative capacity was interrogated by the wound healing assay, wherein the PELI3-deficiency greatly delayed the scratch closure processing (Fig. 2e). Likewise, the invasion was remarkably suppressed by PELI3-silencing as indicated by the decreased invaded cells across BME-coated carbonate membrane (Fig. 2f). In summary, our data highlighted the importance of PELI3 in both proliferative and malignant behaviors of NSCLC.

### Knockdown of PELI3 inhibits tumor growth and metastasis in vivo

**Fig. 3:**
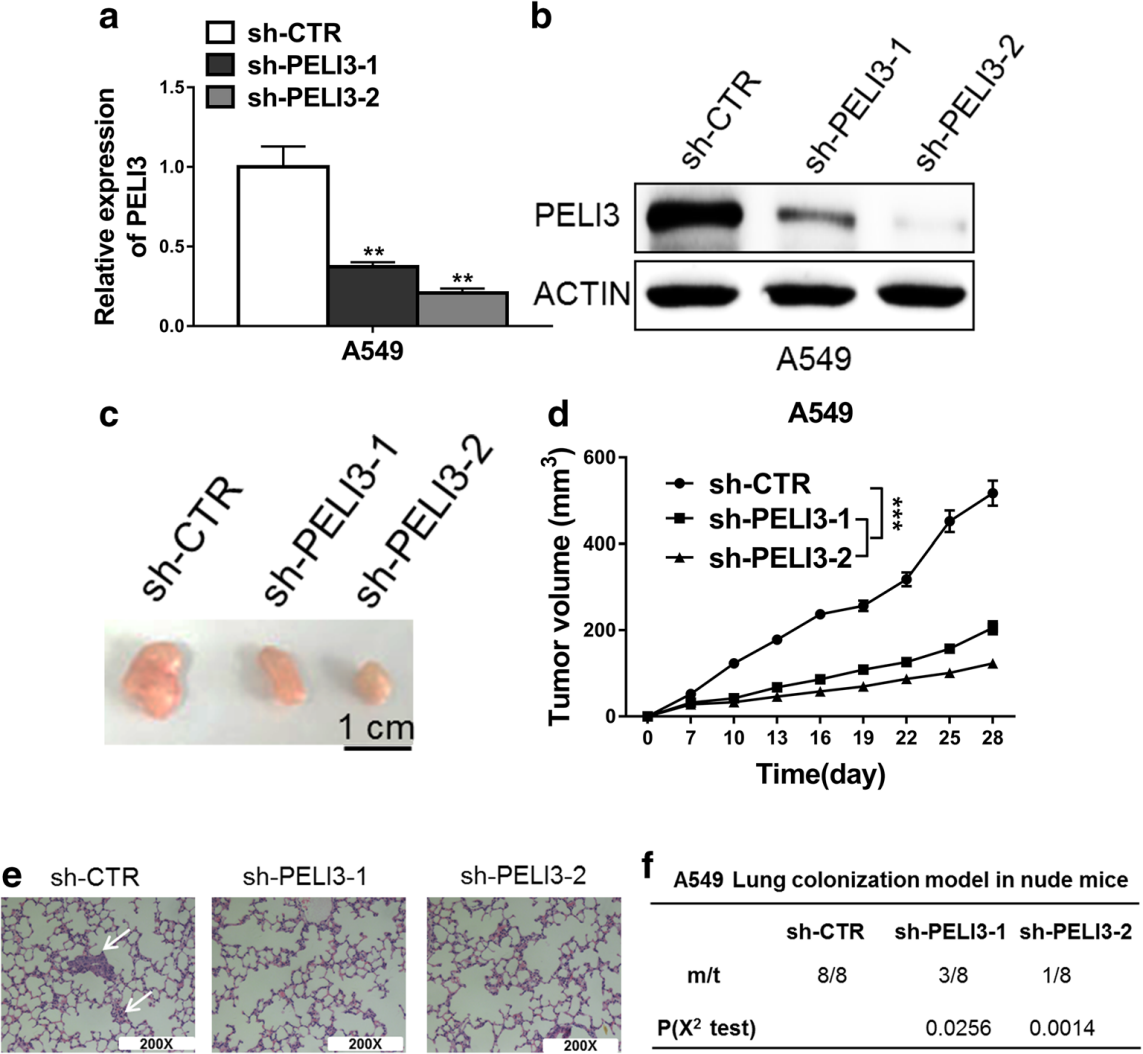
Knockdown of PELI3 inhibits tumor growth and metastasis. **a**, **b** Knockdown efficiency of PELI3 in A549 cells stably transfected with PELI3 shRNA (sh-PELI3-1 and sh-PELI3-2) or empty vector (sh-CTR) was confirmed by qRT-PCR and western blot. **c**, **d** Tumor growth curve of A549-sh-CTR and A549-sh-PELI3 cells implanted into the flank of male nude mice. **e**, **f** H&E staining of the metastatic nodules in the lung of A549 cells which stably transfected with PELI3 shRNA (sh-PELI3-1 and sh-PELI3-2) or empty vector (sh-CTR) following tail vein injection into nude mice (200 × scale bars) and incidence of lung metastasis in mice following tail vein injection of the respective A549 cells. *P < 0.05; **P < 0.01; ***P < 0.001

To exclude the potential artifacts associated with cell culture, we next validated the in vitro observations in xenograft tumor mice. To this end, we established the stable PELI3-deficient cell line in A549, and knockdown efficiency was confirmed at transcript and protein levels by real-time PCR and western blot, respectively (Fig. 3a, b). The xenograft tumor size was significantly decreased in PELI3-deficient group in comparison with control (Fig. 3c) and tumor growth was greatly retarded by PELI3 knockdown during the experimental window (Fig. 3d). Meanwhile, the lung metastasis was monitored by H&E staining with the representative images were shown in Fig. 3e. The metastatic capacities were remarkably inhibited by PELI3 deficiency (Fig. 3f, 8/8 incidence of lung metastasis in control group; 3/8 in sh-PELI3-1, p = 0.0256; 1/8 in sh-PELI3-2, p = 0.0014). Therefore, we consolidated the anti-tumoral effects elicited in PELI3-deficient xenograft tumor model.

### PELI3 is a direct target of miR-365a-5p in NSCLC cells

**Fig. 4:**
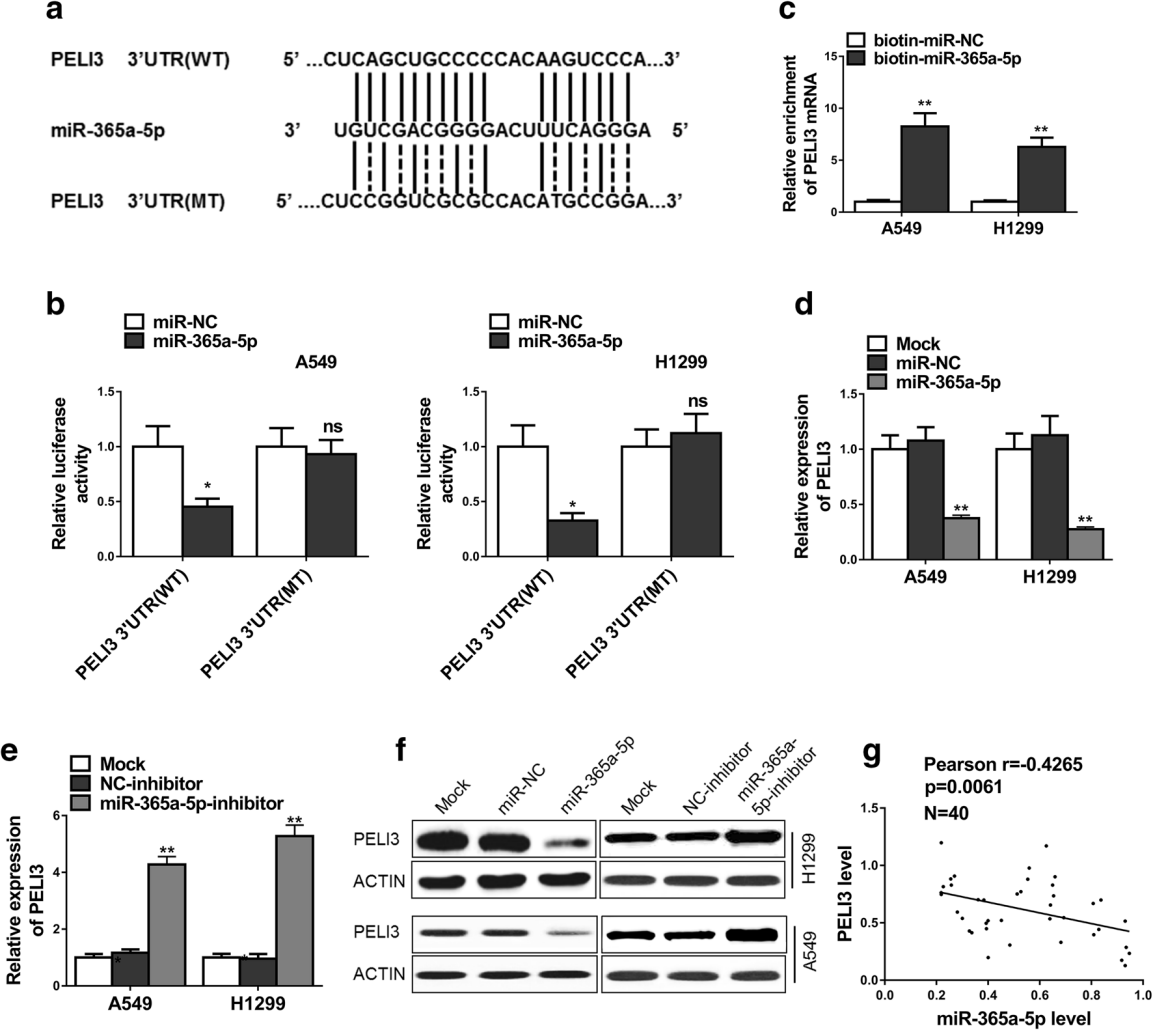
PELI3 is a direct target of miR-365a-5p in NSCLC cells. **a** The wide type (PELI3 3′UTR(WT)) and mutant (PELI3 3′UTR(MT)) miR-365a-5p targeting sequence located in the 3′UTR of PELI3 mRNA. **b** Luciferase reporter assay was performed in A549 and H1299 cells co-transfected with plasmid containing PELI3 3′UTR(WT) or PELI3 3′UTR(MT) and miR-365a-5p or miR-NC. **c** Detection of PELI3 mRNAs in biotinylated miRNA/target mRNA complex by real-time RT-PCR. The relative levels of PELI3 mRNA in the complex pulled down by using biotinylated miR-365a-5p was compared to that of the complex pulled down by using the biotinylated control random RNA. **d**–**f** The relative expression levels of PELI3 in A549 and H1299 cells transfected with indicated microRNA mimics and microRNA inhibitors or their respective negative controls detected by qRT-PCR and western blot. **g** A statistically inverse correlation between PELI3 and miR-365a-5p levels in 40 NSCLC tissues (Spearman’s correlation analysis). The data represent the mean ± SD from three independent experiments. *P < 0.05; **P < 0.01

Next, we sought to elucidate the regulatory mechanism underlying the aberrant over-expression of PELI3 in NSCLC. With aid of bioinformatics algorithm (http://www.targetscan.org/vert_71/), we searched for microRNAs that could target the 3′-UTR of PELI3, and successfully identified miR-365a-5p as a potential candidate. The alignment between miR-365a-5p and 3′UTR region of PELI3 was illustrated in Fig. 4a. The regulatory effect of miR-365a-5p on PELI3 expression in NSCLC was interrogated by the luciferase reporter assay, which was fused to either wild-type or scramble 3′UTR region of PELI3 transcript. Co-transfection with miR-365a-5p into both A549 and H1299 cells significantly suppressed luciferase activity, while scrambled mutation introduced into the putative binding region completely abolished this effect (Fig. 4b). We further confirmed the direct binding between PELI3 transcript and miR-365a-5p using pulldown assay, wherein the PELI3 mRNAs were greatly enriched in the miR-365a-5p pulldown complex other than scramble negative control (Fig. 4c). The endogenous PELI3 was also determined in response to both exogenous miR-365a-5p and its specific inhibitor. The PELI3 transcripts were significantly inhibited while introduced with ectopic miR-365a-5p, while tremendously induced by the miR-365a-5p-inhibitor (Fig. 4d, e), which underlined the fundamental role of miR-365a-5p in regulation of PELI3 expression. This regulatory phenomenon was also validated at protein level in both cell lines (Fig. 4f). Furthermore, the analysis of endogenous expression of PELI3 and miR-365a-5p in NSCLC clinical samples disclosed the reverse correlation between PELI3 and miR-365a-5p transcripts (Fig. 4g). Therefore, we identified that miR-365a-5p as the direct modulator of PELI3 expression in NSCLC.

### MiR-365a-5p is frequently decreased in NSCLC and suppresses proliferation, migration and invasion of NSCLC cell lines

**Fig. 5:**
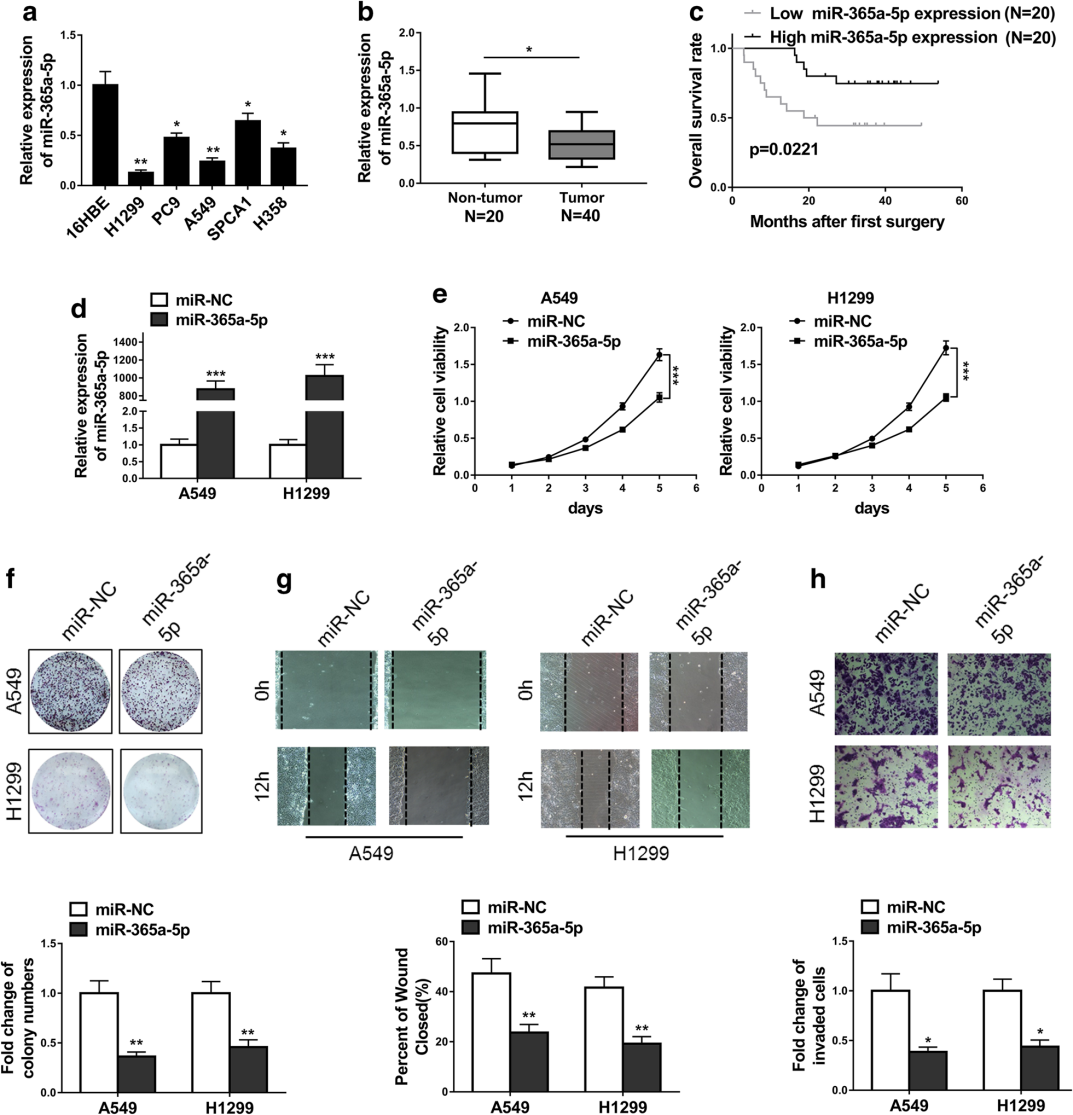
MiR-365a-5p is frequently decreased in NSCLC and suppresses proliferation, migration and invasion of NSCLC cell lines. **a** The expression levels of miR-365a-5p in NSCLC cells (A549, SPCA1, H1299, H358, PC9) and human bronchial epithelial cells (16HBE) were detected by qRT-PCR. **b** The miR-365a-5p expression levels in 40 NSCLC tissues and 20 non-tumor tissues were detected by qRT-PCR. **c** Kaplan–Meier’s analysis of the correlation between miR-365a-5p expression and the overall survival rate of NSCLC patients. **d** The relative expression of miR-365a-5p was quantified by qRT-PCR in A549 and H1299 cells transfected with miR-365a-5p mimic (miR-365a-5p) or respective negative control (miR-NC). **e**, **f** MTT and colony formation assays showed overexpression of miR-365a-5p significantly suppressed proliferation of A549 and H1299 cells. **g** Wound-healing assays showed the repression of cell migration of A549 and H1299 due to overexpression of miR-365a-5p. **h** Transwell assays showed the repression of cell invasion of A549 and H1299 due to overexpression of miR-365a-5p. The data represent the mean ± SD from three independent experiments. *P < 0.05; **P < 0.01; ***P < 0.001

Next, we sought to characterize the expression status of miR-365a-5p in NSCLC and its potential contribution to the tumor biology of NSCLC. The endogenous miR-365a-5p was universally decreased in NSCLC cell lines in comparison with 16HBE cell (Fig. 5a). Likewise, the suppressed expression of miR-365a-5p was observed in the NSCLC clinical samples as well (Fig. 5b). High miR-365a-5p associated with the better prognosis in our clinical pool, which implicated the anti-tumoral properties of miR-365a-5p in this disease (Fig. 5c). The correlative relationship was also observed between miR-365a-5p level and disease progression, wherein 76.5% of patients were miR-365a-5p-positive in stage I–II and only 30.4% with positivity in stage III–IV (p = 0.0095) (Table 2). Next, we ectopically introduced miR-365a-5p into both A549 and H1299 (Fig. 5d). The forced expression of miR-365a-5p significantly compromised the cell viability in both A549 and H1299 cells in our MTT assay (Fig. 5e). Consistent with its anti-tumor properties, ectopic miR-365a-5p greatly decreased the formed colonies in both cell lines (Fig. 5f). In sharp contrast to the stimulatory effect of PELI3, over-expression of miR-365a-5p remarkably delayed the wound healing processing (Fig. 5g) and reduced cell invasion into the BME-coated transwell (Fig. 5h). Our data suggested the anti-tumor activity of miR-365a-5p via antagonism with PELI3.

**Table 2:** Correlation of miR-365a-5p expression with tumor stage in NCSLC

Expression of miR-365a-5p			
Parameter	Low (%)	High (%)	P value
Tumor stage			0.0095
I–II	4 (23.5%)	13 (76.5%)	
III–IV	16 (69.6%)	7 (30.4%)	

### PELI3 mediates the effects of miR-365a-5p on NSCLC cells proliferation, migration and invasion

**Fig. 6:**
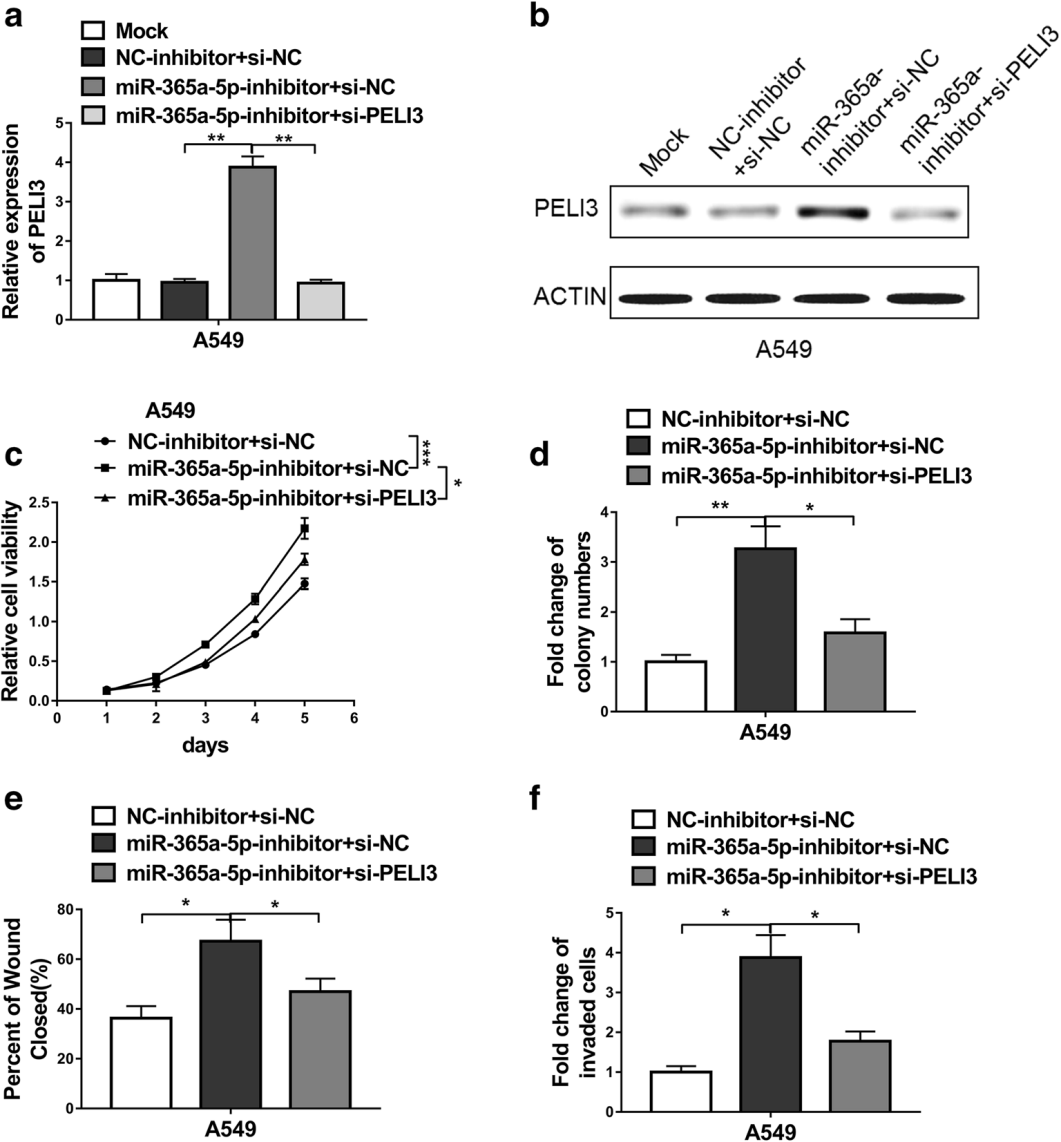
PELI3 mediates the effects of miR-365a-5p on NSCLC cells proliferation, migration and invasion. **a**, **b** The expression levels of PELI3 were measured by qRT-PCR and western blot in A549 cells co-transfected with nothing (Mock), negative control of miRNA inhibitor and negative control of siRNA (NC-inhibitor + si-NC), miR-365a-5p inhibitor and negative control of siRNA (miR-365a-5p-inhibitor + si-NC) or miR-365a-5p inhibitor and PELI3 siRNA (miR-365a-5p-inhibitor + si-PELI3). **c**, **d** MTT and colony formation assays showed cell proliferation advantage induced by miR-365a-5p inhibitor was attenuated by knockdown of PELI3 in A549 cells. **e** Wound-healing assays showed the increased cell migration due to miR-365a-5p inhibitor was attenuated by knockdown of PELI3 in A549 cells. **f** Transwell assays showed the increased cell invasion due to miR-365a-5p inhibitor was attenuated by knockdown of PELI3 in A549 cells. The data represent the mean ± SD from three independent experiments. *P < 0.05; **P < 0.01; ***P < 0.001. (two-way ANOVA for **c**, student’s t-test for others)

Our previous results indicated that PELI3 might function downstream the aberrant over-expressed miR-365a-59 in NSCLC. Next, we sought to experimentally determine the dominance of PELI3 in mediating the anti-tumoral actions of miR-365a-5p in this setting. To this purpose, we specifically silenced PELI3 in the miR-365a-5p-deficient A549 cells and the relative expression of PELI3 was determined by real-time PCR (Fig. 6a). In agreement with previous results, miR-365a-5p inhibition significantly induced up-regulation of PELI3, which was subsequently suppressed by PELI3-specific siRNA (Fig. 6b). The stimulatory effect of miR-365a-5p-silencing on cell viability was completely abolished by PELI3 knockdown (Fig. 6c). Likewise, the remarkable increase of colony formation capacity in the miR-365a-5p-deficient A549 was markedly compromised by downregulation of PELI3 (Fig. 6d). The metastasis related capacities were further assessed in this setting. As shown in Fig. 6e, miR-365a-5p inhibition accelerated the wound closure, which was neutralized by addition of PELI3 siRNA. Similarly, the increased invasive capacity in the miR-365a-5p-deficient A549 was restored by introduction of PELI3-specific siRNA (Fig. 6f). Therefore, our data underlined the dominant role of PELI3 in mediating the anti-tumoral activity of miR-365a-5p in NSCLC.

## Discussion

PELI3 was an important member in the Pellino E3 ubiquitin protein ligase family and recently received attentions in the inflammatory response and insulin resistance [15]. However, its involvement in human malignancies has been barely investigated so far. Here we first analyzed PELI3 expression both in vivo and in vitro. Our data for the first time uncovered the aberrant over-expression of PELI3 in NSCLC at both transcript and protein level. More importantly, high level of PELI3 significantly associated with poorer prognosis of NSCLC patients, which indicated the oncogenic properties of PELI3 in this disease. SiRNA-mediated transient knockdown of PELI3 in both A549 and H1299 cells greatly suppressed cell viability and colony formation capacity. Furthermore, the metastasis-related malignant behaviors such as migration and invasion were compromised by PELI3-silencing as well. We further pursued to address the regulatory mechanisms underlying the aberrant over-expression of PELI3 in NSCLC. The putative candidate miR-365a-5p was identified with bioinformatics online tool. We experimentally demonstrated that miR-365a-5p directly modulated the expression of PELI3 3′UTR-driven luciferase. Our pulldown assay confirmed the direct binding between miR-365a-5p and PELI3 transcript. The endogenous expression of PELI3 was greatly suppressed in response to ectopic introduction of miR-365a-5p, which was subsequently released by miR-365a-5p-inhibitor. Furthermore, the inverse correlation between endogenous PELI3 and miR-365a-5p was observed in NSCLC patients, which underlined the direct regulatory relationship in this scenario. The universal down-regulation of miR-365a-5p in NSCLC was further characterized both in vivo and in vitro. High miR-365a-50 significantly stratified the better survivor from the patients with poor prognosis. Exogenous miR-365a-5p greatly suppressed cell viability and colony formation in A549 and H1299 cells, as well as the wound healing and transwell invasion. More importantly, PELI3-deficiency significantly inhibited cell proliferation, migration and invasion provoked by the miR-365a-5p inhibition, which highlighted the dominant role of PELI3 in mediating the anti-tumor activity of miR-365a-5p in NSCLC.

miR-365a-5p has been increasingly recognized involving in range of human malignancies. For instance, Zhu et al. [16] demonstrated that miR-365 inhibited cell growth and promoted apoptosis in melanoma by targeting Bcl2 and Cyclin D1. Wang et al. [17] showed that miR-365 promoted lung carcinogenesis by downregulating the USP33/SLIT2/ROBO1 signaling pathway. In addition, Min et al. [18] verified that exosomes derived from imatinib-resistant chronic myeloid leukemia cells mediated a horizontal transfer of drug-resistant trait by delivering miR-365. Study by Hamada et al. [19] also disclosed miR-365 induced gemcitabine resistance in pancreatic cancer by targeting the adaptor protein SHC1 and pro-apoptotic regulator BAX. On contrary, the anti-tumor properties of miR-365 were uncovered by vast investigations. Xu et al. [20] proposed that miR-365 functioned as a tumor suppressor by directly targeting CYR61 in osteosarcoma. Chen et al. [21] disclosed the prognostic significance and anti-proliferation effect of miR-365 in hepatocellular carcinoma. Bai et al. [22] provided evidences that miR-365 inhibited growth, invasion and metastasis of malignant melanoma by targeting NRP1 expression. In cutaneous squamous cell carcinoma, Zhou et al. [23] unraveled that miR-365-targeted nuclear factor I/B transcriptionally repressed cyclin-dependent kinase 6 and 4 to inhibit the tumor progression, which was further consolidated by the observation that loss of BAX by miR-365 promoted cutaneous squamous carcinoma progression by suppressing apoptosis [24]. Wang et al. [25] displayed that miR-365 inhibited ovarian cancer progression by targeting Wnt5a as well. In line with the anti-tumoral notion of miR-365, here we provided novel evidences that down-regulation of miR-365a-5p in NSCLC, which further associated with unfavorable outcomes clinically.

Until now, multiple target genes of miR-365a-5p have been experimentally identified. For example, Mori et al. [26] described that HLA-G expression was regulated by miR-365 in trophoblasts under hypoxic conditions. Yang et al. [27] demonstrated that mechanical and IL-1β responsive miR-365 contributed to osteoarthritis development by targeting histone deacetylase 4. Zhang et al. [28] unraveled that miR-365 inhibited vascular smooth muscle cell proliferation through targeting cyclin D1. Wang et al. [29] showed that miR-365 promoted diabetic retinopathy through inhibiting Timp3 and increasing oxidative stress. Wu et al. [30] demonstrated that miR-365 accelerated cardiac hypertrophy by inhibiting autophagy via the modulation of Skp2 expression. Sa et al. [31] reported that post-transcriptional suppression of TIMP-1 in epithelial-differentiated adipose-derived stem cells seeded bladder acellular matrix grafts reduced urethral scar formation. Here our results for the first time identified that PELI3 as the direct target of miR-365a-5p, which consequently contributed to its anti-tumor properties.

## Conclusion

In summary, here we characterized the aberrant down-regulation of miR-365a-5p and sequent up-regulation of PELI3 in NSCLC. Our results suggested the anti-tumoral activity of miR-365a-5p via suppression of PELI3 expression in this disease, which might hold great promise for diagnostic and therapeutic purpose.

## Abbreviations

NSCLC: non-small cell lung cancer
EGFR: epidermal growth factor receptor
RANKL: receptor activator of nuclear factor kappa-B ligand
PELI3: Pellino E3 ubiquitin protein ligase family member 3
MTT: 3-(4,5-dimethylthiazol-2-yl)-2,5-diphenyltetrazolium bromide
